# Measurement of Common Prosperity of Chinese Rural Households Using Graded Response Models: Evidence from Zhejiang Province

**DOI:** 10.3390/ijerph20054602

**Published:** 2023-03-05

**Authors:** Mei Zhang, Xinliang Wang

**Affiliations:** College of Economics & Management, Zhejiang University of Water Resources and Electric Power, Hangzhou 310018, China

**Keywords:** rural households, common prosperity, measurement index, graded response models, validity evaluation

## Abstract

Common prosperity is an important feature of Chinese-style modernization. The difficulty and focus of promoting the construction of common prosperity in China lies in rural areas and rural households. How to evaluate the common prosperity of rural households is becoming an important research topic. Based on the perspective of meeting the people’s needs for a better life, this study designed 14 items or indicators from the dimensions of affluence, commonality, and sustainability. The common prosperity of rural households is regarded as a potential structure. Based on the survey data of 615 rural households in Zhejiang Province, graded response models were used to estimate the discrimination and difficulty coefficient, and an indicator selection and characteristics analysis were carried out. The research results show that there are 13 items suitable for measuring the common prosperity of rural households, and these indicators have strong distinguishing ability. However, different dimension indicators have different functions. In particular, the affluence dimension, the sharing dimension, and the sustainability dimension are suitable for distinguishing families with a relatively high level of common prosperity, a medium level of common prosperity, and a low level of common prosperity, respectively. Based on this, we propose policy recommendations such as building diversified governance strategies, formulating differentiated governance policies, and supporting the corresponding basic policy reform.

## 1. Instruction

Common prosperity is the aspiration and pursuit of many people around the world. However, due to different basic political systems and policy objectives, developed countries and China have obvious differences in the expression and even the connotation of common prosperity. There is no direct expression of “common prosperity” in developed countries, and such concerns are generally attributed to issues of fairness and efficiency [1]. After the Second World War, the welfare economic system, built on the theoretical basis of Keynesianism, emphasized the implementation of high subsidies, high welfare, high taxation, and high protection policies through the realization of full employment, aiming at protecting the weak and restraining the polarization of the rich and poor to a certain extent [2].

The Western industrialized countries began to advocate neoliberalism in the early 1980s because of the serious social problems caused by the simultaneous soaring unemployment and the inflation rate. Its values tend to believe that the wealth gap is a natural phenomenon, the result of market survival of the fittest, and the government should not intervene too much [3]. However, with the deepening of economic globalization, income inequality in developed countries such as the United States, the United Kingdom, and Germany has become increasingly serious. Technological change and globalization are considered to be the key explanatory factors [4]. To alleviate domestic income inequality, Atkinson (2016) advocates the restoration of a more progressive income tax, estate and gift tax, and the imposition of a global corporate minimum tax; the implementation of a basic income system and other interventionist measures [5]. Alacevich and Soci (2018) argue that the expansion and availability of social safety nets is the key to reducing inequality [6]. Wilkinson and Pickett (2019) point out that we should get rid of the concept of status and consumption, promote a democratic employee system, and greatly improve the quality of work [7]. It can be seen that the Western societies also have the social goal of “common prosperity” in their connotation [8].

Unlike Western societies, common prosperity is an essential requirement of socialism with Chinese characteristics and an important feature of Chinese modernization. In particular, since the 18th CPC National Congress, the CPC Central Committee has placed greater importance on achieving common prosperity for all the people. The 19th National Congress put forward the goal of “achieving common prosperity for all”, and the Sixth Plenary Session of the 19th Central Committee emphasized “promoting common prosperity”. “Opinions on Supporting the High-quality Development and Construction of Zhejiang Demonstration Area of Common Prosperity” provides a provincial example for the implementation of common prosperity in the country. The 10th meeting of the Financial and Economic Commission of the CPC Central Committee clarified the general idea of common prosperity. In May 2021, the CPC Central Committee and The State Council officially released the Opinions on Supporting Zhejiang’s High-quality Development and Building a Demonstration Area of Common Prosperity, which provided a provincial example for the implementation of common prosperity in China. At present, China still faces many challenges; there is a wide gap between urban and rural development and between regions, and income distribution. To promote common prosperity, the most arduous and onerous task is in rural areas [9]. There are big differences in pension income between urban and rural areas, and obvious differences in education opportunities and quality between urban and rural areas [10]. The biggest challenge is to narrow the gap between urban and rural areas, and the focus should be on rural residents [11]. The scientific definition and measurement of common prosperity at the micro level of residents has become an important issue to be solved in the new era.

The existing literature discusses the theoretical connotation and measurement indicators of common prosperity extensively. From the theoretical origin, the thought of common prosperity in the new era is the continuous development of the thought of common prosperity of all generations of leaders of the CPC before the 18th National Congress of the Communist Party of China [12]. Common prosperity highlights the political ideal of “seeking the interests of the vast majority of people”, the value orientation of taking the people as the center, and the essence of the new journey of building a modern socialist country [13]. Specifically, some researchers focus on development and sharing. Li and Zhu (2022) believe that common prosperity is a reasonable and differentiated prosperity in which the lowest class of the society can reach the lowest standard of prosperity [10]. Moreover, other researchers emphasize happiness and good life. Liu et al. (2021) pointed out that common prosperity means a multi-dimensional comprehensive happy life and all-round development of human beings [14]. Li (2021) believes that common prosperity means that all the people have various means of production and living to meet their needs for a better life [15]. Liu and Wang (2021) believe that common prosperity means a happy life of shared material prosperity, spiritual self-confidence and self-improvement, social harmony and harmony, and a livable and working environment [16]. According to the institutional design principles, the design of institutional mechanisms and policy systems for common prosperity requires at least two principles: incentive compatibility and institutional matching [17]. Most of the existing literature construct regional common prosperity measurement indicators from a macro perspective, with two dimensions: overall prosperity and development achievement sharing degree [14], and common prosperity and prosperity degree [18]. In addition, some studies also consider that common prosperity includes three-dimensional or four-dimensional indicators. The three-dimensional indicators include basic indicators, core indicators and auxiliary indicators [19], development, sharing, and sustainability, among others [20]. On the other hand, the four-dimensional indicators include popularity, sharing, development, and security [21]. From the perspective of composition, material prosperity and spiritual prosperity have six dimensions: employment and income, social welfare, quality of life, health status, human capital, and spiritual life [22].

As for theoretical connotation of common prosperity of rural households, Du (2022) pointed out that the horizontal dimension of common prosperity mainly includes the level of economic development, material living standards, and cultural living standards [23]. Huang (2022) pointed out that ensuring faster growth of rural household income was the key to achieving common prosperity for all [24]. However, the outstanding problems facing the countryside at present are the more serious relative poverty and vulnerability [25,26,27], poor public service conditions [28], inadequate rural development, unbalanced rural regional development, and asynchronous development of material civilization and spiritual civilization [29]. At the same time, low-income rural households face challenges such as weak or even poor income growth, few and limited ways for sustainable income growth, weak or insufficient income growth ability to become rich, and a large gap from achieving common prosperity [30]. In terms of empirical research, only a few studies have examined the common prosperity of the rural household and its important factors. Zhang et al. (2022) explored the quality of rural life from the perspective of common prosperity [31]. Tan et al. (2022) calculated the temporal and spatial differences of the common prosperity of Chinese rural households at the provincial level by using provincial statistical data [32]. Zhang et al. (2022) measured the common prosperity index at the household level with the analytic hierarchy process and investigated the influence of digital financial inclusion on common prosperity [33]. Liu et al. (2022) measured the common prosperity of rural households by the counting method and verified that social security insurance plays a positive role in improving the common prosperity of rural households [34]. Tan and Wu (2022) adopted the equal-weight scoring method to measure the common prosperity of rural households and found that the flow of the rural labor force significantly promoted the common prosperity of rural households, and the flow within the county area had the greatest effect on the promotion of the common prosperity of rural households [35].

Needless to say, the above research results have a positive reference value and guiding value for the future quantification and path exploration of China’s common prosperity. Most of the existing studies focus on the understanding of regional common prosperity from a macro perspective, and only a few studies pay attention to the common prosperity of rural households. In these literature sources, the measurement concept of multidimensional poverty is used for reference, and the counting method or equal weight scoring method is adopted to measure the common affluence level of rural households, which leads to the reliability of the measurement results. At the same time, the index system of household common prosperity based on value judgment lacks the statistical test of reliability and validity of the index. In contrast to the existing research, we pay more attention to the scientific selection of the measurement method validation of the common prosperity indicators of rural families. Therefore, our research contributions include two aspects. First of all, from the perspective of farmers’ need for a better life, we constructed a micro-level measure of common prosperity in rural areas of Zhejiang Province, which includes income indicators and subjective evaluation indicators in many aspects. However, most of the existing studies used multi-dimensional poverty indicators to examine the micro common prosperity. Second, the graded response model (GRM) is used to select indicators of common prosperity and verify the effectiveness of the selection indicators, which provides a reliable and effective measurement method for other studies, and supplements the shortcomings of the equal weight method.

The rest of the paper is organized as follows: Section 2 introduces the research area and research methods; Section 3 analyzes the GRM results; while Section 4 contains the discussion and Section 5 summarizes the study.

## 2. Materials and Methods

### 2.1. Study Area and Data Source

Common prosperity is an essential requirement of socialism and an important feature of Chinese-style modernization [9]. In October 2020, the Fifth Plenary Session of the 19th Central Committee of the CPC proposed that all people should make more substantial progress with common prosperity [36]. On 20 May 2021, the CPC Central Committee and The State Council officially issued Opinions on Supporting Zhejiang’s High-quality Development and Construction of the Common Prosperity Demonstration Zone, making Zhejiang the first common prosperity demonstration zone in China [37]. The reason is that Zhejiang Province has a high degree of economic prosperity and equilibrium development. The income of urban and rural residents has ranked the first in China for 20 consecutive years and 36 years, respectively, and the income multiple difference between urban and rural residents is only 1.96, far lower than the national level (2.56) [38]. In order to fully implement the decision of the central government, the Zhejiang Provincial government issued the Implementation Plan of Zhejiang High-quality Development and Construction of the Common Prosperity Demonstration Zone (2021–2025) in 2021, proposing provincial model construction purposes such as continuous narrowing of income gap, quality sharing of public services throughout the life cycle, general spiritual prosperity, and social harmony and unity [39]. The Ministry of Agriculture and Rural Affairs has issued the Action Plan to Build a High-quality Rural Revitalization Demonstration Province and Promote the Construction of Demonstration Zones for Common Prosperity (2021–2025), which gives priority to the development of agriculture and rural areas, takes the lead in realizing the integrated development of urban and rural areas, and focuses on narrowing the development gap between urban and rural areas and regions and promoting common prosperity of rural households [40]. The Zhejiang Provincial Department of Agriculture and other departments issued the Action Plan for Promoting Common Prosperity through High-quality Development in Agriculture and Rural Areas (2021–2025), which proposed the specific goals of realizing the “increase of 10,000 yuan” of the income of three agriculture and rural households, the “continuous reduction” of the income double difference of three rural areas, and the “basically doubling” of the governance of three rural areas [41]. It can be seen that the Chinese government attaches great importance to the common prosperity of rural households. In particular, it has put forward specific goals such as “livable and working conditions in rural areas, rich and prosperous rural households, equal access to basic public services in urban and rural areas, and generally satisfying the demands of rural households throughout their life cycle at a higher level”, which provides policy guidance for studying the common prosperity of rural households in Zhejiang.

**Figure 1:**
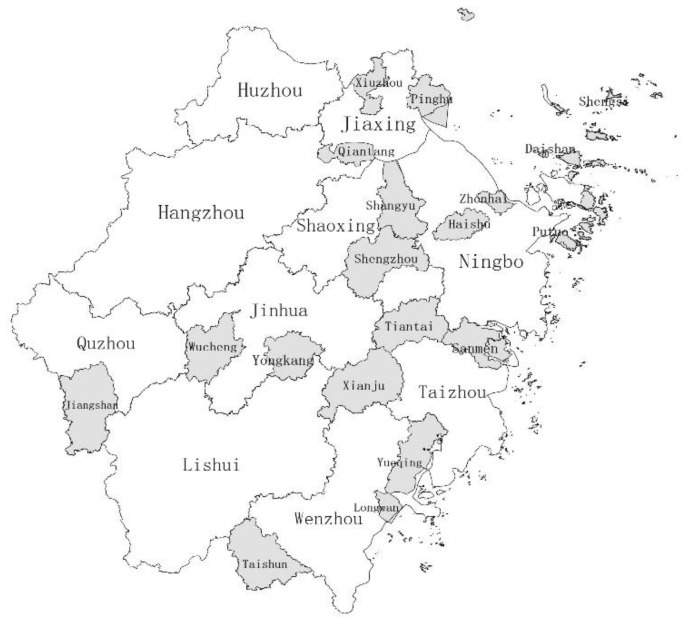
Regional distribution of sample survey in Zhejiang Province, China.

The data are from the rural survey carried out in Zhejiang Province from July to August 2021. The survey adopts the stratified sampling method, including Hangzhou, Ningbo, Jiaxing, Shaoxing, Zhoushan, Quzhou, Jinhua, Taizhou, Wenzhou, among others as shown in Figure 1. According to the size of the urban–rural income multiple difference in 2021, the survey areas were divided into regions with high urban–rural income gap, that is, the urban–rural income multiple difference was greater than 1.8, including Quzhou (1.86), Taizhou (1.92), Wenzhou (1.94), and Jinhua (2.00). In the regions with low income gap, the urban–rural income multiplier difference is between 1.6 and 1.8, including Jiaxing (1.60), Zhoushan (1.61), Shaoxing (1.71), Ningbo (1.72), and Hangzhou Shaoxing (1.75). Twelve villages were investigated in areas with higher income gap and lower income gap respectively, and 20–30 rural households were surveyed in each village. A total of 615 valid questionnaires were obtained, including 307 in areas with high income gap and 308 in areas with lower income gap. Among them, Jiangshan County of Quzhou and Taishun County of Wenzhou are remote mountain villages with lower income level. The contents of the survey include personal characteristics, basic family information, family livelihood strategy and income information, village social and economic information, and the evaluation information of common prosperity index.

### 2.2. Indicators and Dimensions

At the 27th group study session of the Political Bureau of the 19th CPC Central Committee, General Secretary Xi Jinping stressed that common prosperity itself is an important goal of socialist modernization, and we should always take meeting the people’s new expectations for a better life as the starting point and the goal of development [42]. This paper holds that the common prosperity of rural households refers to the abundant condition of the good life of all the people under the socialist condition, which is compatible with the modernization of economy and society. Chen (2022) believes that common prosperity is always relative to “people’s needs”, and satisfying people’s needs is a truly scientific and human-oriented natural measure to assess common prosperity [43]. From the perspective of constituent connotation, the essence of common prosperity is to meet people’s ever-growing needs for a better life in an all-round way, including material life needs, spiritual and cultural needs, basic public services needs, and ecological needs of a better livable environment [44].

**Figure 2:**
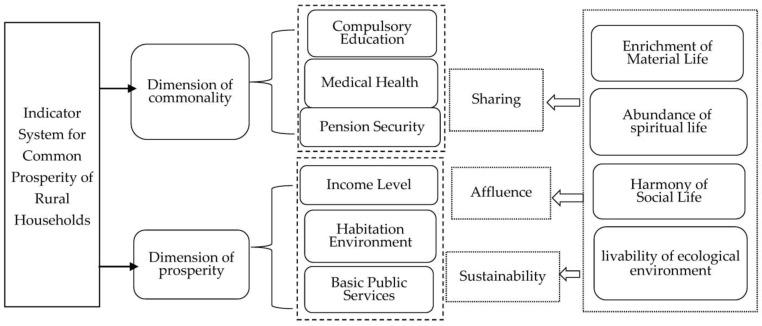
Analysis framework of common prosperity of rural families in China.

Therefore, the common prosperity of rural households is a comprehensive unity of material life, social life, spiritual life, and livable ecological environment (Figure 2). From the perspective of evaluation indicators, “commonality” and “prosperity” are important dimensions to measure common prosperity [17,20]. It reflects the scope of rural household prosperity, that is, households enjoy equal development opportunities and achievements. The sharing index is used to measure the following, namely: (1) Compulsory education: average education level of the labor population, satisfaction with compulsory education, and evaluation of the gap between urban and rural education, (2) Medical and health care: self-health evaluation, evaluation of the reimbursement ratio of basic medical insurance, satisfaction of basic medical insurance for urban and rural residents, (3) pension security: satisfaction of basic pension insurance for urban and rural residents.

Prosperity reflects the relative abundance of rural households’ average living standard, including prosperity and sustainability. The prosperity index measures the affluence of the material life of rural households. It adopts five equal points of per capita income, whether the per capita income of the household is less than 50% of the median income of the sample, income satisfaction, per capita income gap with the per capita income of urban residents, and other indicators. Sustainability indicators measure the sustainability of rural household’s living environment and access to basic public services. The following indicators are used: (1) Living environment—garbage disposal evaluation, village security evaluation, sanitary toilet construction evaluation; (2) Public service opportunities—evaluation of cultural entertainment places, fitness facilities, convenience of medical treatment, and service evaluation of village committees.

For all the items, the data given in our study are ordered categories, and their values are 1–5. In order to provide more detailed information, Table 1 provides descriptive statistics of mean and standard deviation for the common prosperity indices of rural households.

### 2.3. Analytical Statistical Method

Before investigating the common prosperity of rural households, reliable measure indicators should be determined. Given that the commonly used classical measurement methods cannot provide sufficient information for the measurement attributes of each single indicator, we use a more reasonable item response theory (IRT) to test the reliability and effectiveness of indicators. The item response theory is widely applied to scale evaluation in education and psychology research, and is also being applied to economic and social science research, such as poverty [45,46], and women’s empowerment [47]. Our analysis uses the graded response model [48], which can process the data of grade and continuous response. Compared with the classic testing theory, IRT has many advantages. First, items and trait parameters are relatively invariable. Second, it defines items and test information functions that are not in the classical theory. It provides independent reliability index information for each item, taking into account the matching of item difficulty level and subject level [49]. However, IRT has some limitations. For example, it is based on complex mathematical models and relies on stronger assumptions. In addition, it requires more strict test conditions, larger sample size, and more items. If these conditions are not met, the estimation accuracy may be inaccurate.

In the GRM, common prosperity can be viewed as a latent construct, measured by a series of explicit indicators. Suppose that for the *i* th household (*i* = 1, 2…*n*) and *j* th test items (*j* = 1, 2…m), all items take on the ordered categories (*k* = 0, 1, …, *K*), *a_j_* is the discrimination parameter of the *j* th test item, *b_jk_* is the *k*th cutpoint for item *j*, or is considered as the difficulty of responding with category *k* or higher for item *j*, *Y_ij_* is the answer of the *i* th household to the *j* th test item, *θ_i_* is the latent trait ability parameter of the *i* th household, which conforms to normal distribution and represents the common prosperity degree of the household.

GRM is specified with respect to the probability that the response will be observed in category *k* or higher [50], is composed of cumulative probabilities, and its specific function form is as follows:

(1) $$P\left(Y_{ij}\geq k|\theta_{i}\right)=\frac{exp\left\{\alpha_{j}\left(\theta_{i}-b_{jk}\right)\right\}}{1+eexp\left\{\alpha_{j}\left(\theta_{i}-b_{jk}\right)\right\}}\;\;\;\;\theta_{i}\sim N\left(0,1\right)$$

To further calculate the probability of selecting *k* when the *i* th rural households answers the *j* th question, the formula is as follows:

(2) $$P\left(Y_{ij}=k|\theta_{i}\right)=P\left(Y_{ij}\geq k|\theta_{i}\right)-P\left(Y_{ij}\geq k+1|\theta_{i}\right)$$

In addition, on the basis of parameter estimation, we use Bayesian expectation posterior estimation method to estimate the common prosperity of families *θ I* value. The estimation formula is as follows:

(3) $$E\left(\theta_{n}|Y_{n}\right)=\overset{\infty }{\underset{-\infty }{\int }}\theta_{n}\left\{\overset{I}{\underset{i=1}{\prod }}P\left(y_{in}|\theta_{n}\right)\right\}g\left(\theta_{n}\right)d\theta_{n}$$

## 3. Results

### 3.1. Hypothesis Test

Before the parameter estimation, IRT requires the unidimensionality and monotonicity of response hypothesis test. In the paper, we used factor analysis to carry out the unidimensionality test. First, we tested the reliability and validity of the items. The result of Cronbach’s Alpha Index showed that Cronbach’s α of all items was 0.890, Cronbach’s α of the first level was 0.729, 0.864, and 0.894 respectively. Kaiser–Meyer–Olkin and Bartlett’s spherical test was used for validity testing, and the results showed that Cronbach’s α was greater than 0.7, the internal consistency of the item was credible. At the same time, the overall KMO was 0.889, and the KMO value of each item ranged from 0.546 to 0.964.

In addition, the significance of the statistical value of Bartlett’s sphericity test is 0.000 (χ^2^ = 5778.71). Therefore, factor analysis was suitable for our research. The result showed that the characteristic value of the first factor is 6.380, the characteristic value of the second factor is 1.669, and their ratio is 3.820. From the above results, the single dimension of the trait space is established. In addition, Mokken scale procedure (MSP) is used to verify monotonicity. Specifically, MSP is based on Loevinger’s H value [51], and higher positive Loevinger’s H values reflect better appropriateness of the scale. Mokken (1971) suggested that the borderline value of the Loevinger’s H is 0.30 [52]. Table 2 results show that the Loevinger’s H values of six items are all more than 0.3, i.e., the 14 items are consistent with the monotonicity assumption.

### 3.2. Analysis of Parameter Estimation Results

In the GRM, the reasonable value range of the discrimination parameter a and the difficulty coefficient b is an important basis for item evaluation. The discrimination a reflects the discrimination degree of the item to the ability level of the subjects. The higher the value of a, the stronger is the discrimination ability of the item. According to the study [53], the value of the discrimination parameter a is between 0.3 and 4). On the other hand, the difficulty coefficient b reflects the difficulty of the item. The higher the value of b, the greater is the difficulty of the item. Based on the research [54], we believe that the difficulty of the item is moderate when the value range of b is in the range of −4 to 4.

Table 3 lists the results of parameter estimation. The result shows that only item 2 has a discrimination of less than 0.3, the discrimination parameter of item 1 and item 3 to item 14 is between 0.331 and 3.670, both greater than 0.3. Therefore, these items have good distinction at the trait level. Further analysis found that the discrimination and difficulty coefficient of item 2 exceeded the interval value, and the item should be deleted. At the same time, the difficulty coefficients of item 4 to item 14 are between −3.966 and 2.216. The change of the estimated values of the difficulty parameters of different categories of each item shows that the common wealth level of bj2–bj5 is distributed from low to high, monotonically increasing, and there is no inverse threshold.

It should be noted that the difficulty coefficient of item 1 is only slightly less than negative 4 when the division point is negative 2, which is close to the standard value. The difficulty coefficient of item 1 is only slightly greater than 4 when the division point is 2, which is close to the standard value. In this case, the item 1 can be retained. Similarly, the difficulty coefficient of item 3 is only slightly less than −4 when the division point is 2, which is close to the standard value. Therefore, item 4 can be retained.

**Figure 3:**
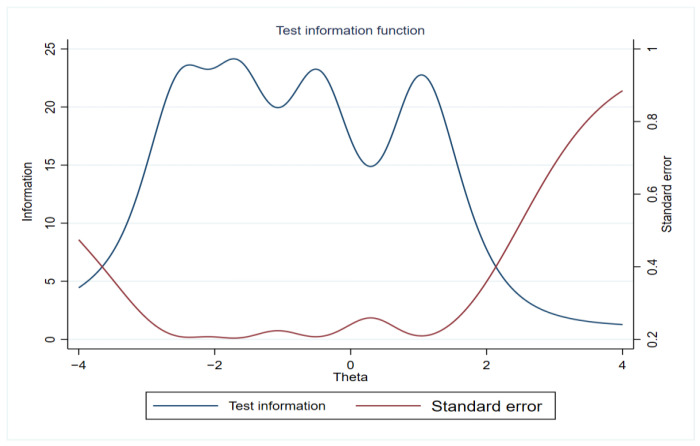
Test information function.

Figure 3 shows the test information curve, providing the total information amount and measurement error. Specifically, when the common-wealth level is about −3.5, and 2, the test information value is higher than 6.67, reflecting a good reliability result. In general, the discrimination parameters of the items used are relatively good, and the measurement accuracy is also relatively high.

**Figure 4:**
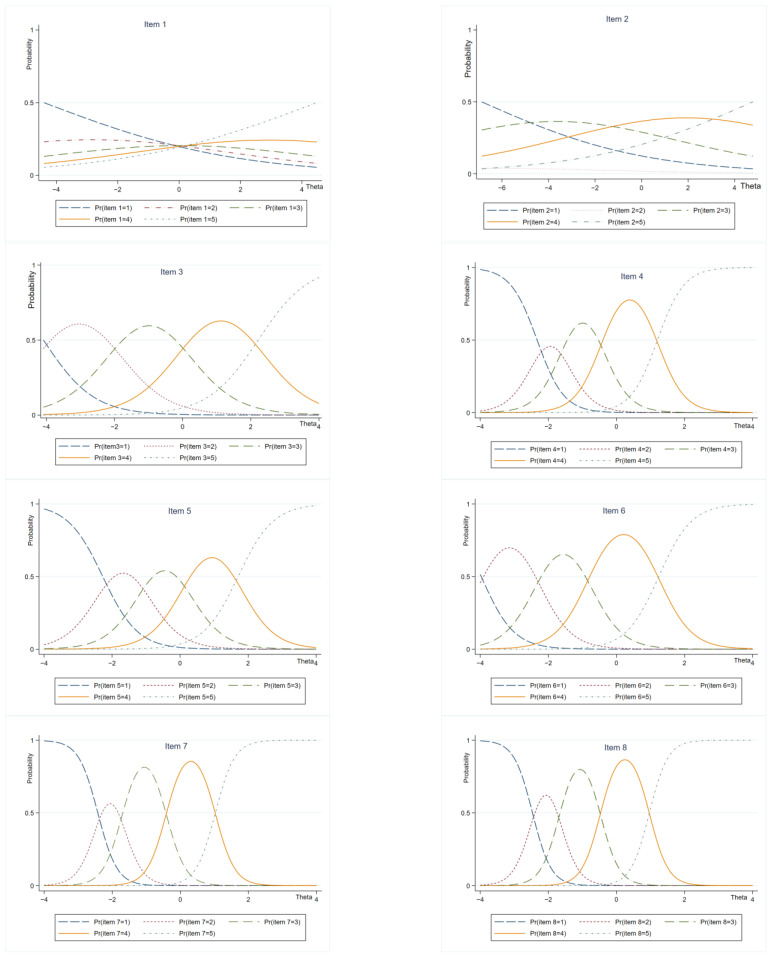

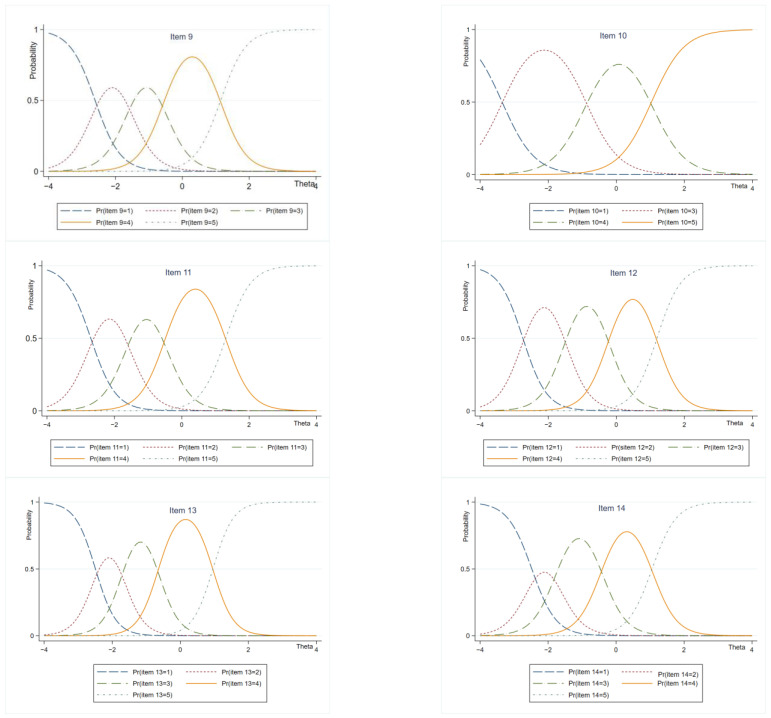
Item information function matrix.

Figure 4 is the matrix diagram of the project characteristic curve (ICC) of each item. Under ideal conditions, the first and fifth curves of ICC change monotonously, and the second and fourth curves are normal distribution. The results show that the ICC curve distribution of items 1 and 2 is not ideal, and the ICC curve of other items is ideal.

### 3.3. Analysis of Family and Regional Characteristics

On the basis of parameter estimation, the Bayesian expectation posteriori estimation method is used to calculate the theta value of the common wealth potential variable of rural households. Table 4 lists the results of theta mean value of the group. From the perspective of gender, the level of common prosperity of male-headed households is higher than that of female households. In terms of age, the level of common prosperity of households headed by households under 30 years old is the lowest, because these families are at the stage of establishment, and their income ability, wealth level, and social participation ability are relatively weak. However, the level of common prosperity of nuclear families or trunk families (the head of household is from 31 to 65 years old) is relatively higher. From the perspective of family size, the level of common prosperity of families with more than five people is the lowest, and that of families with less than three people is relatively higher. From the perspective of education level, the education level of the head of household is the highest among the families in high school, and the common prosperity level of other families is similar. It is worth noting that the level of common prosperity of families headed by college degrees is not high. The possible reason is that these families are a small number of relatively young heads of households who choose to return to rural life and start businesses after graduation from college. They are at the beginning of their careers. Now the level of common prosperity is low but there is more room for the growth of the level of common prosperity in the future. From the perspective of livelihood strategy, the common prosperity level of business households is higher than that of migrant families. Compared with the family income level, the common prosperity level of families with a total income of more than 150,000 yuan is relatively higher. From the perspective of regional differences, the level of common prosperity of families living in areas with higher urban–rural income difference is lower, while the level of common prosperity of families living in areas with lower urban–rural income difference is higher. In general, these conclusions are basically in line with the expectations.

## 4. Discussion of Findings

Some studies use survey data to measure the level of common prosperity after setting the indicators of common prosperity of rural households directly through value judgment. They use the analytic hierarchy process [33], the counting method [34], and the equal weight scoring method [35] to measure the common prosperity of rural households. However, there may be two defects. The first is lack of testing the reliability and effectiveness of the set indicators, and the direct measurement of the level of common prosperity cannot provide scientific evidence of indicators. Second, the multi-dimensional poverty calculation method is adopted to assign 0 and 1 values to the common prosperity indicators. The equal weight method may ignore the fact that different indicators have different importance to rural households, such as the importance of education and income to different rural households. In view of this, this paper uses the item response theory (IRT) to conduct an in-depth study of the differentiation and difficulty coefficient of the measurement indicators, and provide methods and evidence support for the future micro-level common wealth measurement and indicator optimization.

For the Dimension of Affluence, items from 1 to 2 respectively represent the per capita income level and income satisfaction of families. Their function is to distinguish families with relatively high level of common prosperity. However, the discrimination ability of income satisfaction items is high. The research conclusion is similar to that of Tan et al. [32]. We consider that the income factor is the material basis for the common prosperity of rural households, and the sustained growth of income and the failure to narrow the income gap between urban and rural areas represents the basic conditions for promoting the common prosperity of rural households. The dimension of wealth is an important aspect of evaluating the common prosperity of rural households. For the Dimension of Sharing, the discrimination ability of items from 4 to 8 is higher than that of the Dimension of Affluence, and the discrimination coefficient of these items is almost higher than 2. However, the indicators reflecting education, medical care, old-age care, and other factors are only suitable for distinguishing families with middle level of common prosperity. For farmers with middle level of affluence, on the basis of meeting their basic material needs, they pursue better life needs such as education, old-age care, and medical care, so these indicators can better reflect their common prosperity. This is different from the study of Tan et al. [32]. For the Dimension of Sustainability, items from 9 to 14 have strong discrimination ability, and the discrimination coefficient of these items is higher than 2. However, these items reflecting rural living environment and public service opportunities are suitable for distinguishing families with a low level of common prosperity, which is different from the study of Tan and Wu [35]. For farmers with a relatively low level of common prosperity, they live in remote mountainous areas in Zhejiang Province, the garbage treatment, sanitary toilets, cultural and entertainment facilities, and medical conservation facilities in the village are relatively backward. Therefore, meeting these ideal needs is an important factor to achieve their common prosperity.

From the individual, family and regional characteristics of common prosperity, the level of common prosperity of male households is higher than that of female households; The larger the population, the lower the level of common prosperity, which is the same as the conclusion of Zhang et al. [33]. Although Tan and Wu (2022) found that the head of household with higher education is conducive to improving the level of common prosperity [35], we consider that the level of common prosperity of families who return to their hometown to start businesses after graduation from university is not necessarily higher than that of other families in rural Zhejiang.

## 5. Conclusions

This study designed the measurement indicators of the common prosperity of Chinese rural households from the dimensions of wealth, common prosperity, and sustainability, and applied the survey data of rural households in rural areas of Zhejiang Province, using the discrimination and difficulty coefficient estimated by the GRM model to examine the reliability and effectiveness of selecting the indicators of common prosperity of rural households. We found that only one item did not meet the requirements, and the other 13 items could be used to examine the common prosperity of Chinese rural households. However, different dimension indicators have different functions. The dimension of affluence item representing family income level and willingness can be used to distinguish families with relatively higher common prosperity, and the dimension of sharing items representing education, medical care, old-age care, among others can be used to distinguish families with medium level of common prosperity. The dimension of sustainability item, which reflects the rural living environment and public service opportunities, is suitable for distinguishing families with lower common prosperity. Therefore, in the process of promoting the common prosperity of rural households, we should pay more attention to the indicators of the dimension of sharing and the dimension of sustainability, which are more important for middle and lower common prosperity families.

According to the above research conclusions, three policy implications are obtained. First, we should understand in depth the theoretical connotation of common prosperity and build diversified governance strategies. We should not only continue to broaden the channels for farmers to increase their income, but also ensure their income stability. For example, actively promoting the in-depth reform of the rural property rights system and the income distribution system of rural residents and realizing the organic synergy between the income growth and income distribution of rural residents [55]. We should also improve the quality of rural education, medical care, old-age care, other public services [56], improve rural garbage disposal, sanitary toilets and other infrastructure, and gradually improve rural medical and health conditions [57]. Second, we should implement differentiated governance policies according to people’s needs. For instance, for people with a lower level of common prosperity, rural industry revitalization and farmers’ income and quality improvement should be carried out on the basis of improving village-level infrastructure and public service supply. On the other hand, for people with medium wealth level, we should focus on improving the quality of basic public services such as education, medical care, and old-age care through policy coordination. Third, the corresponding basic policy reforms should be carried out to provide institutional guarantee for the overall common prosperity of rural farmers. For example, carry out the basic distribution system, reform the rural collective economic system [23], build a policy support system for the common development of large farmers and small farmers [24], weave and tighten the rural subsistence security system [30], and promote the equalization of basic public services in urban and rural areas.

Despite the findings outlined in this paper, our research has several limitations. First, researchers have not reached a consensus on the measurement indicators of the common prosperity of micro-families, and their scope is relatively wide. However, the 14 indicators designed in this paper are not comprehensive enough and need to be further expanded. Second, this paper only surveys Zhejiang Province, the demonstration area for the construction of common prosperity in China, without considering rural families in other regions of China. In addition, we only use cross-sectional data, and the results of individual, family and regional characteristics of common prosperity of rural households are not suitable for causal inference. Finally, we did not consider the differential item function in the method, which may affect the accuracy of the research results to a certain extent. In future, we propose to build indicators of common prosperity at the national level and carry out large-scale research to support the implementation of these indicators at the operational level. At the same time, we should continue to learn from the experience of Zhejiang Province in the promotion of common prosperity to achieve common prosperity for farmers across the country.

## Figures and Tables

**Table 1:** Descriptive statistics for the common prosperity indices of rural households.

Dimensions (Latent Variable)	Measurement Index	Value Range	Mean (Standard Deviation)
Dimension of Affluence	Grouping of household per capita income (Item 1)	1–5	3.000 (1.415)
	Grouping of urban–rural income gap (Item 2)	1–5	3.507 (1.212)
	Subjective evaluation of income satisfaction (Item 3)	1–5	3.444 (0.813)
Dimension of Sharing	Satisfaction degree of compulsory education (Item 4)	1–5	3.649 (0.882)
	Evaluation of urban–rural education gap (Item 5)	1–5	3.250 (0.990)
	Evaluation of self-health (Item 6)	1–5	3.859 (0.709)
	Satisfaction with reimbursement of medical insurance (Item 7)	1–5	3.730 (0.794)
	Satisfaction of basic endowment insurance (Item 8)	1–5	3.763 (0.792)
Dimension of Sustainability	Satisfaction with village waste treatment (Item 9)	1–5	3.737 (0.836)
	Satisfaction with village security (Item 10)	1–5	3.958 (0.688)
	Satisfaction with sanitary toilet construction (Item 11)	1–5	3.701 (0.806)
	Satisfaction with cultural and entertainment facilities (Item 12)	1–5	3.605 (0.844)
	Satisfaction with medical convenience (Item 13)	1–5	3.829 (0.777)
	Satisfaction of village committee and village affairs (Item 14)	1–5	3.725 (0.822)

**Table 2:** Loevinger’s H value of 14 items.

Item 1	Item 2	Item 3	Item 4	Item 5	Item 6	Item 7
0.379 ***	0.303 ***	0.403 ***	0.454 ***	0.416 ***	0.414 ***	0.506 ***
Item 8	Item 9	Item 10	Item 11	Item 12	Item 13	Item 14
0.502 ***	0.457 ***	0.448 ***	0.459 ***	0.488 ***	0.490 ***	0.458 ***

Note: *** are significant at 1% levels.

**Table 3:** Parameter estimation results of GRM.

Items	Discrimination Coefficient (ai)	Cut- Point	Difficulty Coefficient (bi)	Items	Discrimination Coefficient (ai)	Cut- Point	Difficulty Coefficient (bi)
Income (Item 1)	0.331 *** (0.075)	≥2	−4.251 *** (0.986)	Satisfaction with endowment (Item 8)	3.670 *** (0.291)	≥2	−2.460 *** (0.222)
		≥3	−1.214 *** (0.373)			≥3	−1.665 *** (0.100)
		≥4	1.305 *** (0.374)			≥4	−0.467 *** (0.055)
		≥5	4.322 *** (0.987)			≥5	0.962 *** (0.078)
Incomes gap group (Item 2)	0.284 *** (0.075)	≥2	−6.870 *** (1.842)	Satisfaction with garbage (Item 9)	2.629 *** (0190)	≥2	−2.611 ** (0.222)
		≥3	−6.325 *** (1.699)			≥3	−1.581 *** (0.101)
		≥4	−0.958 *** (0.388)			≥4	0.550 *** (0.061)
		≥5	4.779 *** (1.280)			≥5	1.153 *** (0.091)
Satisfaction with Income (Item 3)	1.348 *** (0.113)	≥2	−4.089 *** (0.443)	Satisfaction with safety (Item 10)	2.083 *** (0.161)	≥2	—
		≥3	−1.998 *** (0.167)			≥3	−3.350 *** (0.363)
		≥4	0.040 (0.078)			≥4	−0.879 *** (0.078)
		≥5	2.216 *** (0.180)			≥5	1.026 *** (0.093)
Satisfaction with Education (Item 4)	2.552 *** (0.179)	≥2	−2.326 *** (0.171)	Satisfaction with toilet (Item 11)	2.688 *** (0.194)	≥2	−2.701 ** (0.238)
		≥3	−1.552 *** (0.098)			≥3	−1.593 *** (0.101)
		≥4	−0.420 *** (0.059)			≥4	−0.493 *** (0.059)
		≥5	1.199 *** (0.093)			≥5	1.318 *** (0.095)
Education gap (Item 5)	1.959 *** (0.138)	≥2	−2.258 *** (0.168)	Satisfaction with facilities (Item 12)	2.849 *** (0.201)	≥2	−2.745 ** (0.254)
		≥3	−1.073 *** (0.084)			≥3	−1.497 *** (0.093)
		≥4	0.173 *** (0.067)			≥4	−0.224 *** (0.056)
		≥5	1.700 *** (1.120)			≥5	1.202 *** (0.090)
Satisfaction with health (Item 6)	2.083 *** (0.155)	≥2	−3.966 *** (0.580)	Satisfaction with convenience (Item 13)	3.3217 ** (0.253)	≥2	−2.493 ** (0.209)
		≥3	−2.301 *** (0.172)			≥3	−1.689 *** (0.104)
		≥4	−0.810 *** (0.073)			≥4	−0.643 *** (0.059)
		≥5	1.239 *** (0.102)			≥5	0.963 *** (0.080)
Satisfaction with insurance (Item 7)	3.546 *** (0.277)	≥2	−2.419 *** (0.201)	Satisfaction with public opinion (Item 14)	2.796 *** (0.202)	≥2	−2.481 ** (0.199)
		≥3	−1.699 *** (0.103)			≥3	−1.743 *** (0.111)
		≥4	−0.410 *** (0.054)			≥4	−0.423 *** (0.058)
		≥5	1.023 *** (0.081)			≥5	1.066 *** (0.086)

Note: ** and *** are significant at 5%, and 1% levels respectively. Standard error is in brackets. “—” indicates no value.

**Table 4:** Analysis on the characteristics of rural households’ common prosperity.

Variables	Theta	Variables	Theta	Variables	Theta
Male	**0.620**	4–5 people	0.593	Income ≥ 100,000 Yuan	0.568
female	0.587	More than 5 people	0.577	Income ≤ 150,000 Yuan	**0.622**
≤30 years old	0.582	Primary school education	0.592	Income ≤ 200,000 Yuan	**0.633**
31~45 years old	**0.611**	Junior high school education	0.596	Income > 200,000 Yuan	**0.621**
46~65 years old	**0.621**	High school education	**0.661**	Areas with high income ratio	0.591
Over 65 years old	0.596	College degree	0.593	Areas with higher income ratio *	0.558
≤2 people	**0.615**	Migrant families	0.581	Areas with lower income ratio	**0.620**
3 people	**0.640**	Business family	**0.622**	Sample mean	0.605

Note: Theta is the normalized value. Asterisk refers to the value after deducting the lower income ratio (1.86) of Jinhua City in 2021. Bold indicates exceeding the sample mean.

## Data Availability

No applicable.
